# Disturbing the activity of the primary motor cortex by means of transcranial magnetic stimulation affects long term memory of sentences referred to manipulable objects

**DOI:** 10.3389/fpsyg.2023.1175217

**Published:** 2023-06-30

**Authors:** Francesca Vitale, Manuel de Vega

**Affiliations:** Instituto Universitario de Neurociencia (IUNE), Universidad de La Laguna, La Laguna, Spain

**Keywords:** transcranial magnetic stimulation (TMS), language, memory, embodied cognition, motor system

## Abstract

**Introduction:**

Previous studies on embodied meaning suggest that simulations in the motor cortex play a crucial role in the processing of action sentences. However, there is little evidence that embodied meaning have functional impact beyond working memory. This study examines how the neuromodulation of the motor cortex (M1) could affect the processing of action-related language, measuring participants’ performance in a long-term memory task.

**Method:**

Participants were submitted to two sessions in separate days, one with low-frequency repetitive transcranial magnetic stimulation (rTMS) and the other with sham rTMS. The pulses were delivered for 15 minutes over M1 or over V1, used as a control area. After each stimulation or sham period, the participants were asked to memorize a list of simple sentences, with a manual action verb or an attentional verb, followed in both cases by a noun referred to a manipulable object (e.g., to hang a cane vs. to observe a cane). Finally, they received the verbs as cues with instructions to recall the nouns.

**Results:**

The results showed that low frequency rTMS on M1, compared to sham stimulation, significantly improved the performance in the memory task, for both types of sentences. No change in performance was found after the rTMS stimulation of V1.

**Discussion:**

These results confirm that the perturbation on the motor system, affect the memory of manipulable object names in the context of sentences, providing further evidence of the role played by the sensorimotor system in the encoding and recall of concrete sentences of action.

## Introduction

Which is the causal role of the motor cortex in long-term memory of language referred to manipulable objects? The embodied approach to language processes has devoted especial attention to the processing of action-related sentences, revealing that the motor cortex plays a role during their comprehension (Gallese and Lakoff, 2005; Barsalou, 2008; Fischer and Zwaan, 2008; García and Ibáñez, 2016). Thus, neuroimaging (Tettamanti et al., 2005; Raposo et al., 2009; de Vega et al., 2014), EEG (van Elk et al., 2010; Moreno et al., 2013, 2015), non-invasive brain stimulation (Oliveri et al., 2004; Gerfo et al., 2008; Papeo et al., 2009; Candidi et al., 2010; Repetto et al., 2013; Gianelli and Volta, 2014; Vukovic et al., 2017; Vitale et al., 2021), and brain patients studies (Bak et al., 2001; Desai et al., 2015; Birba et al., 2022) have provided extensive evidence that the motor and premotor cortex (PMC) are activated for action language, especially referred to hand actions, but not for abstract or attentional verbs. However, those studies generally show the intervention of the motor cortex during comprehension or its impact on the immediate recall of information.

The use of transcranial brain stimulation to perturb the activity of a brain region and observe changes in the performance of cognitive tasks could provide evidence for the causal role of the primary motor cortex (M1) in the comprehension of action language. Thus, in some studies participants receive 1-Hz rTMS offline on M1 before performing a semantic or morphological task with action and non-action words (Gerfo et al., 2008; Repetto et al., 2013). Given the inhibitory effect of low-frequency rTMS, performance was selectively impaired (slowing responses) for action words, both verbs and names. In the same vein, online high-frequency rTMS applied to M1 slowed down responses in a semantic judgment task for action related words (Vukovic et al., 2017) and increased the amplitude of the ERP component N400, which is a reliable index of semantic processing, in a hand-related words priming paradigm (Kuipers et al., 2013).

These studies are not entirely conclusive, however, because most of them tested the functional impact of M1 on performance in an immediate task, typically while the received action verb is still active in working memory. However, some behavioral (Dutriaux and Gyselinck, 2016; Dutriaux et al., 2018) and EEG studies (de Vega et al., 2021) on embodied meaning suggest that motor simulations play a crucial role in the long-term memory of nouns in the context of action verbs. These studies showed that holding the hands behind the back interferes with the memorization of manipulable object presented as pictures or as words, but does not affect recall of non-manipulable objects or words (Dutriaux and Gyselinck, 2016); the hands behind back posture also interfered with the memory of action sentences compared to attentional sentences (Dutriaux et al., 2018). In addition, an EEG study using the same memory paradigm, found suppression in fronto-central beta rhythm desynchronization, a brain signature of motor processes, when participants held the hands-behind-the-back posture, compared with the hands in front posture, but only during the processing of manual action sentences (de Vega et al., 2021). Taken together, all these findings suggest the key role of neural motor simulation as a functional aspect of memory for action language.

However, to our knowledge, there is only one study that examines the impact of M1 neuromodulation on long-term memory (Vitale et al., 2021). In that study the participants received in two separate sessions offline sham and active tDCS on M1. At the end of each session, they were given sentences referred to manipulable objects in the context of action verbs or attentional verbs, and after a distractive task, the participants were cued with the verbs and asked to recall the missing names. The results showed that after excitatory stimulation of M1 (anodal tDCS) memory performance selectively improved for names in the context of action verbs. The study clearly demonstrated a causal impact of M1 activity on long-term performance, beyond working memory. However, some limitations of the tDCS techniques should be notice. First, ordinary tDCS involves large electrodes that according to computational estimations produce diffuse electric fields in the brain, which means that other regions beyond the target M1 could be stimulated (Mikkonen et al., 2018, 2020); second, the tDCS induced electric fields varied considerably among individuals because of their individual cranial and brain anatomy (Laakso et al., 2019).

Most of the literature on action-related language focuses on the comparison between manual action verbs (e.g., write, clap), and non-action verbs (think, remember), reporting behavioral facilitation or interference on actions performed with the same effector (compatibility effects) for the former, suggesting that motor cortex activity is involved. Thus, behavioral studies have shown language-action compatibility effects (Shebani and Pulvermüller, 2013, 2018; Zhang et al., 2016). For instance, performing a complex rhythmic hands movement impairs working memory for hand-related words (e.g., clap), while rhythmic feet movements led to selectively impairs memory for foot-related words (e.g., kick) (Shebani and Pulvermüller, 2013, 2018). However, nouns of manipulable objects (pen, hammer) can be also considered action related words, given the implicit motor affordances of the referred objects, which may share neural processes with action verbs (Stanfield and Zwaan, 2001; Glenberg and Kaschak, 2002; Bub et al., 2018). For instance, a neuroimaging study demonstrated that naming manual actions and naming manipulable objects share neural activations in fronto-parietal networks that subserve hand action representation (Saccuman et al., 2006), and a study with single pulse TMS on hand-related M1 revealed that nouns of manipulable objects modulate corticospinal excitability (Gough et al., 2012). In addition, inhibitory neuromodulation on M1 increases reaction times in a concrete/abstract judgment task both for manual action verbs and manipulable object nouns (Gerfo et al., 2008). Finally, Parkinson’s disease patients (PD) and controls were asked to give a bottom-pressing response to pictures or names of graspable and non-graspable objects. Whereas the controls showed interference for graspable objects/nouns (slower responses) indicating underlying motor processing, PD patients produced similar response times for graspable and non-graspable objects (Buccino et al., 2018). In sum, manipulable object names might trigger motor cortex activity and therefore could be sensitive to neuromodulation of M1.

The current research examined the role of the motor cortex on long term memory for action sentences, using the same materials and task demands as in Vitale et al. (2021) study; that is, participants initially read a set of sentences composed of manipulable object nouns presented in the context of either action or attentional verbs (learning phase); then, in the testing phase, they were asked to recall the object nouns that were associated with action or attentional verbs, using a cued recall procedure. However, unlike in Vitale et al., in this research we employed repetitive transcraneal magnetic stimulation (rTMS), which has better accuracy and focality than the tDCS used by those authors. This is an important decision, as the spatial precision of TMS is extremely high, especially when M1 is the target region, and TMS is combined with electromyography to obtain motor threshold (MT) for a single muscle. In contrast, tDCS is imprecise as it does not adapt to the individual anatomy of skulls and brains. Furthermore, the electric field induced by TMS is quite focal, while the electric field induced by tDCS is diffuse and other regions beyond the target can be stimulated (Mikkonen et al., 2018, 2020; Laakso et al., 2019). In one stimulation group, rTMS was applied on the target region M1 to directly test its functional role in language comprehension. To ensure full control conditions, all participants were tested in two sessions, one with active rTMS and the other with sham rTMS, which served as a baseline control session. In addition, a second control-stimulation group was introduced, in which the stimulation was administered on V1, which has no functional relationship with the task. Therefore, we expect that M1 stimulation will modulate long term memory performance, whereas V1 stimulation will not.

A low frequency (1 Hz) rTMS protocol was chosen in the experiment, which is often considered “inhibitory.” So, rTMS might be expected to reduce the excitation of M1 and thus impair long-term memory for action language, indicating a functional relationship. However, the direction of the effects may be reversed in some circumstances, as some memory studies have reported enhanced recall for pictures and words after 1 Hz-rTMS in the DLPF cortex (Turriziani et al., 2012; Patel et al., 2020; van der Plas et al., 2021). Furthermore, offline inhibitory theta burst targeting PMC was shown to enhance the performance of action verbs in a subsequent lexical decision task (Willems et al., 2011). Although the task demand and target region stimulated here are different from those used in that study, it is possible that 1 Hz-rTMS over M1 could also improve performance for a long-term memory task. By contrast, the stimulation on V1 would not change performance, confirming the specific functional role of M1 on long-term memory of action related language. We selected V1 as the control region because it is a standard protocol used by previous studies (Boggio et al., 2008; Makoshi et al., 2011; Avenanti et al., 2012; Casula et al., 2014), and because it is a region not involved in the language processing network.

Finally, the modulation of performance could be constrained to the retrieval of object names learned in the context of action verbs, as previously reported after M1 stimulation (Vitale et al., 2021), or when the hands posture was manipulated (Dutriaux et al., 2018; de Vega et al., 2021). Another possibility is that the retrieval of names is modulated by M1 stimulation regardless of the type of verb in the learning context. This possibility derives from the fact that the names always referred to manipulable objects, which are action words with motor affordances (Gerfo et al., 2008; Dutriaux and Gyselinck, 2016).

## Material and method

### Participants

Sixty Spanish speaking students took part in the study (mean age ± SD: 19 ± 2). All the participants were right-handed, without visual or medical problems, or contraindication to TMS (Rossi et al., 2009, 2011; Rossini et al., 2015). The participants were randomly assigned to two different stimulation group: 30 participants (5 men, mean age ± SD: 19 ± 1) were assigned to the M1 group, where the TMS stimulation was applied over M1 and 30 (5 men, mean age ± SD: 19 ± 2) participants were included in the control group, in this case the target site of the stimulation was V1.

### Linguistic material

The material consisted of 30 action verbs, 30 attentional verbs and 120 manipulable objects combined to create to two set of 120 Spanish simple sentences, with “verb + article + noun” syntax. Within each set, each verb appeared twice, associated with a different object, which were only presented once. The material was organized in a way that if a noun was associated with a manual verb in set 1 (e.g., *colgar un bastón*/to hang a cane), then, in set 2, it was associated with an attentional verb (e.g., *observar un bastón*/to observe a cane) and vice versa. The material was the same used in a previous study (Vitale et al., 2021), and was considerably validated, ensuring that there are no differences in frequency, length and familiarity between the action and attentional verbs (see Table 1). The two types of verbs differed in concreteness, since the manual action verbs were judged as more concrete than the attentional verbs, reflecting the expected semantic differences between them.

**Table 1:** Scores of psycholinguistic variables and statistics for manual action and attentional verbs.

	Action	Attentional	*t*	*p*-level
Frequency	23.83 ± 54.91	56.84 ± 93.87	−1.66	0.10
Length	6.67 ± 1.37	7.27 ± 1.87	−1.41	0.16
Familiarity	6.24 ± 0.52	5.97 ± 0.76	1.55	0.13
Concreteness	5.72 ± 0.51	4.28 ± 0.63	9.77	<0.001**

** denote a strong significant comparisons.

Furthermore, we made sure that the sentences were semantically comparable for both sets (see Vitale et al., 2021 for a detailed description of the validation analysis). The results indicate that the number of verb-object co-occurrences for manual action sentences did not differ from the number of co-occurrences for attentional sentences (set 1: manual action = 31,762 ± 55,992, attentional = 78,917 ± 343,442; t118 = −1.05, *p* = 0.30; set 2: manual action = 88,682 ± 310,006, attentional = 115,036 ± 409,065; t118 = −0.40, *p* = 0.69).

### rTMS parameters and site localization

**Figure 1:**
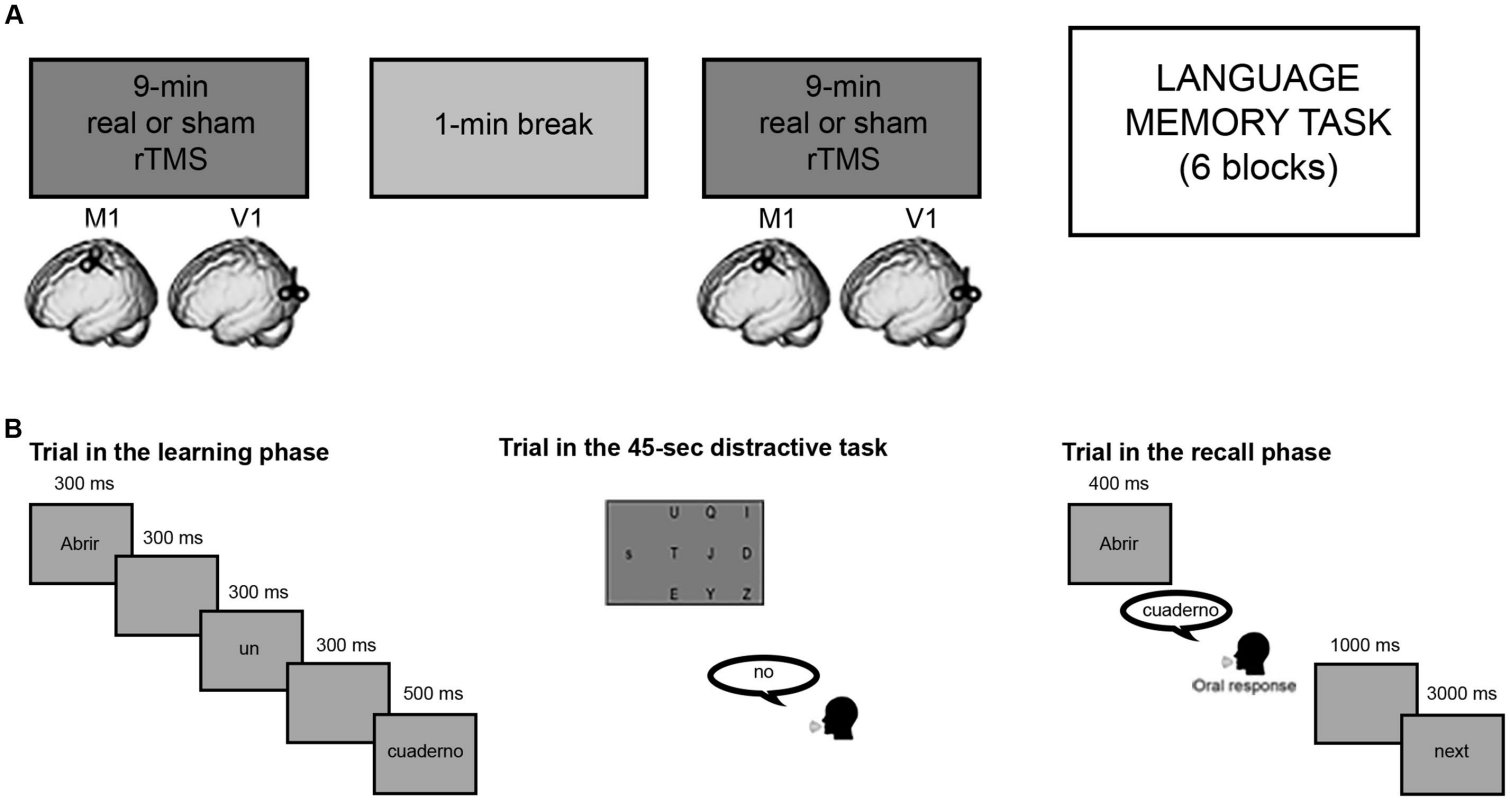
Experimental structure. **(A)** Representation of offline stimulation protocol before the behavioral task. **(B)** Language memory task structure: example of a trial in the learning phase (translation: to open/a/notebook), example of a trial in a distractive task, example of a trial in the recall phase.

Low-frequency rTMS was applied through a figure-of-eight coil connected to a Magstim Rapid2 stimulator (Magstim, Whiteland, Dyfed, United Kingdom). In the M1 group, active stimulation was delivered over the hand representation in the left M1. The exact location was defined as the point where stimulation consistently evoked the largest motor-evoked potentials (MEPs) in the right first dorsal interosseous muscle (FDI). The intensity of stimulation was set at 80% of the resting motor threshold (rMT), defined as the minimal intensity of stimulator output that evoked MEPs with an amplitude of at least 50 μV in the FDI in a series of 10 stimuli (Rossini et al., 2015). With a frequency of 1 Hz, inhibitory rTMS was given in two separate blocks of 9-min stimulation, separated by 1-min break during which overheated stimulation coils were changed (see Figure 1A). In the active stimulation the coil was placed tangentially to the skull, with the handle orientated posteriorly at 45° angle from the midline, while, in the sham condition, the coil was placed vertically over left M1, oriented at 90° with respect to the scalp. The latter stimulation allows to replicate the auditory and somatosensory effect of real TMS, but without inducing any stimulation in the brain.

In the control group, participants underwent the same experimental procedure of the M1 group, but in this case the locus of stimulation was V1, located 3 cm anterior and 1 cm lateral from the inion (Silvanto et al., 2007; Casula et al., 2014). The coil was held with the handle pointing upwards and the stimulation protocol was the same as in the M1 group, with the difference that the stimulation intensity was set at 60% of maximum stimulator output (Silvanto et al., 2005).

### Procedure

The experiment was programmed using Python software. In each stimulation group, all participants were tested in two different sessions, separated by at least 7 days. In the active session, real rTMS was delivered over the target region immediately before the participants performed the memory task, while, in the sham session, the task was preceded by sham stimulation. The order of the sessions was counterbalanced across participants.

The memory task consisted of 6 blocks for each session, for a total of 12 blocks. Each block consisted of a learning phase, followed by a distractive task and a final recall phase (Figure 1B). In the learning phase, participants were asked to memorize as many sentences as possible, as they would be required to recall them later. Each block in the learning phase always began with a filler sentence, not tested in the recall phase to avoid primacy effects, followed by 10 sentences (5 manual action and 5 attentional) appearing in random order. The sentences were presented word-by-word separated by a 2-s intertrial interval, which allowed minimizing mental rehearsal of the sentence that had just been read. Immediately after the learning phase, participants were submitted to a 45-s distractive task, consisting of perceptual letter-matching trials, in which they had to respond whether a target lower-case letter on one side of the screen, was among nine upper-case letters presented on another part of the screen (Dutriaux and Gyselinck, 2016; de Vega et al., 2021). This task was intended to avoid recency effects, as well as working memory strategies such as mental rehearsal or top-down semantic elaboration of learned sentences (e.g., Ye and Xiaolan, 2007; Rose et al., 2014). In the recall phase following the distractive task, participants were given the verbs of the previous sentences as memory cues and had to orally recall the object names associated with them. Responses were recorded and analyzed offline.

### Data analysis

Memory accuracy, calculated as the percentage of objects correctly recalled, was analyzed with a linear mixed model, using the lme4 package in R (Bates et al., 2015). Group (M1, V1), Type of Sentences (action, attentional) and Session (sham, active) were defined as fixed factors, while participants were accounted for as a random effect in the model. Post-hoc analysis were conducted using the false discovery rate (FDR) (Benjamini and Hochberg, 1995) to correct for multiple comparisons.

### Result

The linear mixed model showed a strong main effect of Type of Sentences (F_1,174_ = 29.56; *p* < 0.0001; η*p*^2^ = 0.15), driven by a better memory for manual action sentences (34% ± 16%) relative to attentional sentences (28% ± 17%). As shown in Table 2, such difference between type of sentences in the retrieval of manipulable object names appears in all the experimental conditions, independently of stimulation session (active or sham) and stimulation group (M1 or V1).

**Table 2:** Mean and standard deviation of memory accuracy for the action and attentional sentences, after receiving sham or active stimulation, in both M1 and V1 groups.

	M1 stimulation		V1 stimulation	
	Sham	Active	Sham	Active
Action sentences	30% ± 14%	35% ± 13%	36% ± 21%	35% ± 20%
Attentional sentences	24% ± 15%	30% ± 16%	28% ± 20%	29% ± 20%

**Figure 2:**
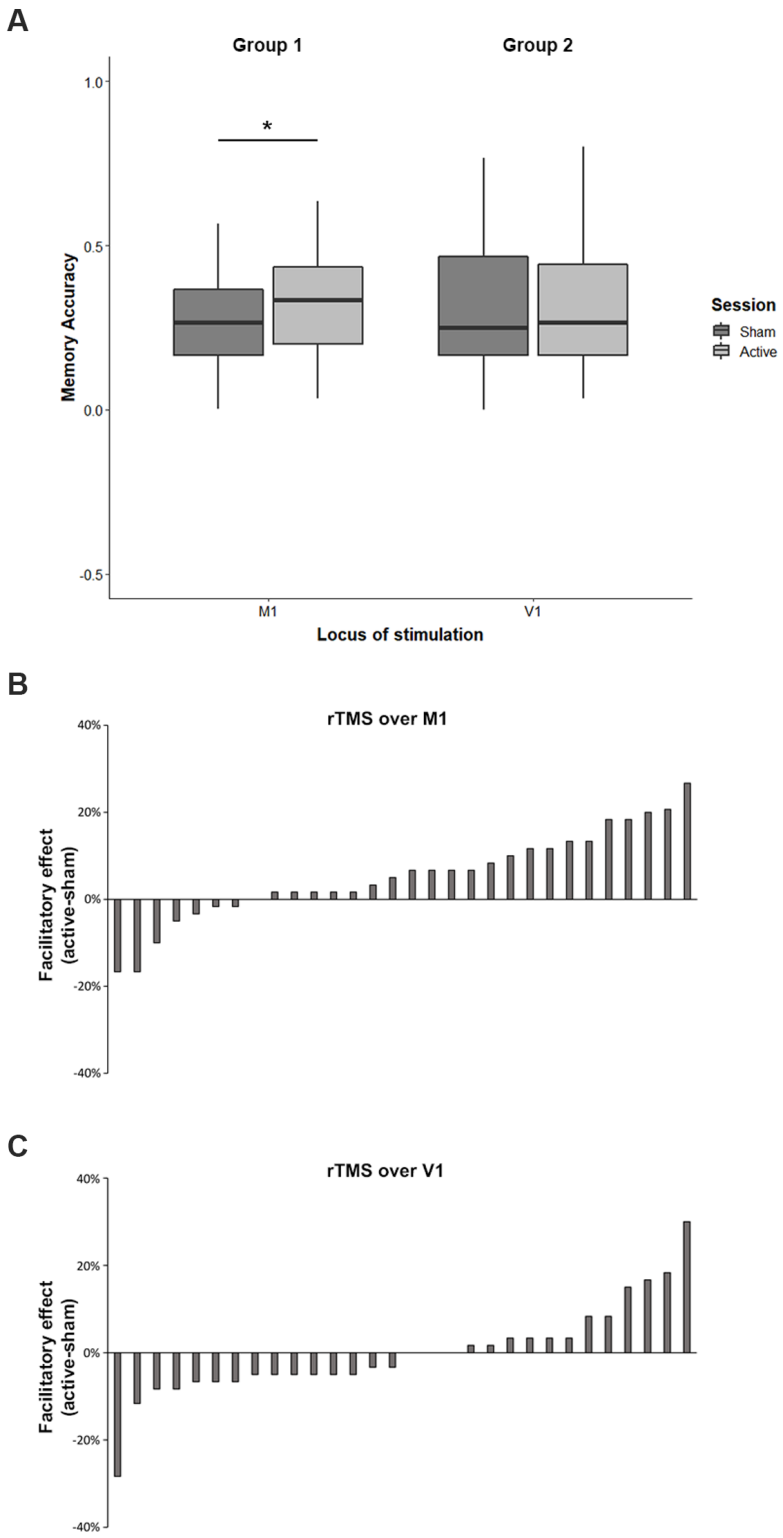
Effect of active stimulation on memory performance in M1 and V1 (left and right of the panel, respectively). Error bars indicate standard error of the mean (SEM). ∗*p* < 0.05 **(A)**. Changes in recall across participants (difference between active and sham rTMS) in M1 **(B)** and in V1 **(C)**.

More importantly, the analysis revealed a significant Group x Session interaction (F_1,174_ = 4.70; *p* = 0.03; η*p*^2^ = 0.03). As shown in Figure 2A, in the experimental group, whose target area was M1, recall of both sentences was significantly better after the active rTMS session (33% ± 13%) relative to sham session (27% ± 12%, *p* = 0.01, Cohen’s *d* = 0.47). On the contrary, as we expected, in the control group, stimulating V1, no changes were found. That is, participants’ performance did not improve when they received active rTMS over V1 (32% ± 19%) compared to the sham stimulation (32% ± 19%). Figure 2B shows the changes in recall, expressed as the difference in accuracy between active and sham stimulation in M1, across participants, where positive and negative values indicate improved and impaired performance, respectively. As can be seen, the facilitatory effects after rTMS on M1 were obtained in 23 participants out of 30. On the other hand, Figure 2C shows that the stimulation of V1 produced more variable results, with a more symmetric distribution centered at zero, with 15 participants showing impairment and 12 participants showing improvement in the recall of manipulable object.

## Discussion

This study demonstrated that focal neurostimulation of M1, by means of offline rTMS, modifies performance in a cue-recall task in which object names were retrieved in response to verb cues. Specifically, rTMS on the motor cortex improved recall for manipulable object names, learned in action verb and attentional verb contexts. The results were robust, and their validity was guaranteed by the strict control conditions of the experimental design. That is, there was a within-participants control, which consisted of testing performance in two sessions under active and sham stimulation, respectively. In addition, a control group was introduced, in which a non-motoric region (V1) was stimulated. Only active rTMS on M1 induced changes in recall, demonstrating a functional role of this region in memory for manipulable object names, and supporting the general idea that embodied representations play a causal role in long-term memory of sentences.

*The recall of objects names depends on the type of verb context*. Before commenting the impact of neuromodulation on memory, let us consider the main effect of verb: the names are always better recalled in action verb contexts than in attentional verb contexts, independently of the stimulation protocol, confirming previous results in the literature with similar materials and task demands as ours (Dutriaux and Gyselinck, 2016; Dutriaux et al., 2018; de Vega et al., 2021; Vitale et al., 2021). Since the names are the same in both contexts, this should be explained by some feature of the verbs, such as their differences in concreteness (see Table 1) or the fact that action verbs are multimodal (e.g., visual and motor) recruiting more extensive neural networks than attentional verbs. Another possibility is that the name of manipulable objects is more predictable in the context of action verbs than in the context of attentional verbs, because the associative strength verb-object is larger in the former than the later.

*Cortical inhibition improved performance*. Previous research has shown that 1 Hz-rTMS on M1 inhibits or reduces cortical excitability (Maeda et al., 2000; Houdayer et al., 2008; Hoogendam et al., 2010, see Fitzgerald et al., 2006 for a review). If so, we might expect impaired performance in tasks that depend on M1, such as action related language. However, we found the opposite, that is, an enhanced memory of manipulable object names after receiving inhibitory rTMS. This paradoxical effect is not new in the literature of memory. For instance, 1 Hz rTMS applied over DLPF, although it reduces the activation of this region, improves working memory and episodic memory performance (Turriziani et al., 2012; Patel et al., 2020; van der Plas et al., 2021). There is even a study in which inhibitory theta burst applied on PMC improved performance on a lexical decision task with action verbs (Willems et al., 2011). Thus, we can tentatively state that reducing M1 excitability could induce facilitatory effects on memory for action related language, as has been frequently reported in other studies of memory with verbal and nonverbal materials. However, the specific computational mechanisms at the neuron level and functional connectivity, by which the reduction of cortical excitability can improve memory, have not yet been established in the literature. The development of neurocomputational approaches could provide a better comprehension of some paradoxical effects frequently found in the embodied semantic literature (Pulvermüller, 2013, 2018).

*Neuromodulation improves names recall in action and attentional contexts.* Contrary to our expectations, the rTMS on M1 enhanced recall of manipulable names in both action verb and attentional verb contexts. In previous studies with similar materials and task demands, the hands-back posture at learning (Dutriaux et al., 2018; de Vega et al., 2021) or the offline excitatory tDCS on M1 (Vitale et al., 2021) selectively modulated memory performance for action verb contexts, either impairing or improving retrieval, respectively. So, why did we not replicate the selective effect of the verb? Here, stimulating the motor cortex with high-precision and focal inhibitory rTMS improved retrieval in both verb contexts, and this was a robust pattern shared by most participants (23 out of 30, see Figure 2B), compared to the diffuse effect and high inter-individual variability of tDCS neuromodulation (Evans et al., 2022, see Li et al., 2015; Vergallito et al., 2022 for reviews). As we mentioned earlier, action sentences are systematically better remembered than attentional sentences in all conditions, indicating that some differential property of the verbs induced this result, since the object names associated with the verbs were the same. In turn, the fact that recall improved after rTMS for the two sentence types can only be attributed to features of the of the shared objects. In other words, focal and intensive activation in M1 acts upon the motor representations of object names, which seems to increase their associative strength with contexts, whether they are action verbs or attentional verbs, improving cue-based recall in both cases. The best explanation for this undifferentiated impact of stimulation comes from the features of the target words to be recalled, which unlike in many other studies on embodied language, were manipulable object names, which were shared by both action and attentional contexts. Manipulable object names are action words that may themselves activate motoric brain regions. Thus, manipulable object names exhibit motor compatibility effects in behavioral experiments (Zhang et al., 2016; Buccino et al., 2018), share motor cortex activations with action verbs in neuroimaging studies (Saccuman et al., 2006), induce corticospinal modulations when TMS is applied in M1 (Gough et al., 2012), show similar changes of performance on a semantic task as action verbs after rTMS in M1 (Gerfo et al., 2008), and reduce recall when learning takes place in hand-behind-back posture (Dutriaux and Gyselinck, 2016).

In conclusion, this research supports the functional link between the activity of the motor cortex and the performance in a long-term memory task for action language. Specifically, the offline modification of M1 excitability by means of focal and high-precision rTMS determined an improvement in the memory of manipulable object names learned both in the context of action verbs and attentional verbs. The affordances of the names, presumably associated with the activity of motoric brain networks, could be responsible of this overall improvement in recall after M1 stimulation. The study reinforces the embodiment semantic approach by demonstrating a brain-memory causal link for action language.

*Limitations.* Despite the contribution of this study, it has some limitations. First, here we focus on the impact of low-frequency rTMS exclusively in M1, yet other areas of the motor network, in particular the PMC, contribute significantly to action language comprehension (Hauk and Pulvermüller, 2004; Pulvermüller, 2013, 2018). Indeed, modulation of PMC activity affects performance on some language tasks, specifically those involving verbs and sentences with motor content (Willems et al., 2011; Tremblay et al., 2012; Gijssels et al., 2018). Future complementary studies could assess whether PMC plays also a role in memory for manipulable objects. Second, due to high inter-individual variability in response to rTMS, and because the direction rTMS-induced behavioural effects was unexpected, physiological data, such as cortico-spinal excitability measured by TMS-induced MEP, should be recorded before and after the stimulation. This could allow assessment of whether TMS is actually inhibiting M1 activity in each participant.

## Data availability statement

The raw data supporting the conclusions of this article will be made available by the authors, without undue reservation.

## Ethics statement

The studies involving human participants were reviewed and approved by Comité de Ética de la Investigación y de Bienestar Animal, Universidad de La Laguna. The patients/participants provided their written informed consent to participate in this study.

## Author contributions

FV analyzed and interpreted the data, wrote the original draft, and provided funding. MV conceptualized the study, supervised the research at all stages, and provided funding. All authors contributed to the article and approved the submitted version.

## Funding

This work was supported by grants from the Spanish Ministerio de Ciencia e Innovación and the European Regional Development Funds (grant PID2021-126172NB-I00) awarded to MV and the Research Training Predoctoral grant (BES-2016-078438) to FV.

## Conflict of interest

The authors declare that the research was conducted in the absence of any commercial or financial relationships that could be construed as a potential conflict of interest.

## Publisher’s note

All claims expressed in this article are solely those of the authors and do not necessarily represent those of their affiliated organizations, or those of the publisher, the editors and the reviewers. Any product that may be evaluated in this article, or claim that may be made by its manufacturer, is not guaranteed or endorsed by the publisher.
